# Is the SPLUNC1-Orai1 axis a critical determinant of lung health?

**DOI:** 10.1042/BST20241029

**Published:** 2025-06-30

**Authors:** Robert Tarran

**Affiliations:** Division of Genetic, Environmental and Inhalational Disease, Department of Internal Medicine, Kansas University Medical Center, Kansas City, KS 66160, U.S.A.

**Keywords:** BPIFA1, calcium channels, ELD607, Orai1, SPLUNC1

## Abstract

Short palate lung and nasal epithelial clone 1 (SPLUNC1; gene name *BPIFA1*) is a secreted protein that is highly expressed in the nasopharyngeal and pulmonary systems. By data mining, we found that SPLUNC1 is also expressed in other organs, including the kidneys and the pituitary gland. SPLUNC1 is an asthma and cystic fibrosis gene modifier that also inversely correlates with the severity of bronchiectasis. Orai1 is a plasma membrane Ca^2+^ channel that is an essential regulator of the immune system. We previously found that SPLUNC1 binds to Orai1, causing it to be ubiquitinated, internalized and trafficked to the lysosome for degradation, thus reducing Ca^2+^ signaling. Here, we discuss how dysregulation of SPLUNC1–Orai1 interactions may contribute to hyperinflammation in multiple pulmonary diseases. We, and others, have also targeted Orai1 therapeutically, and we will also discuss how Orai1 inhibition may overcome SPLUNC1 deficiency and be beneficial for the treatment of chronic lung disease.

## SPLUNC1 nomenclature

Short palate lung and nasal epithelial clone 1 (SPLUNC1) has experienced a checkered naming history. It was originally identified by Weston et al. and named ‘Palate Lung and Nasal Epithelial Clone’ (PLUNC) based on its expression profile [1], but when other family members were identified, it became *‘short* PLUNC1’ (SPLUNC1). While the SPLUNC1 nomenclature remains commonly used for functional studies, its gene name was changed from ‘PLUNC’ to ‘Bacterial Permeability Increasing Protein Family member 1’ (BPIFA1) to reflect its perceived similarity to ‘Bacterial Permeability Increasing Protein’. To further complicate the matter, SPLUNC1 was independently discovered and named both ‘Secreted Protein from the Upper Respiratory Tract’ and ‘Lung Specific Protein-X’ [2,3]. However, I will refer to it as SPLUNC1 in this review.

## SPLUNC1 structure

**Figure 1:**
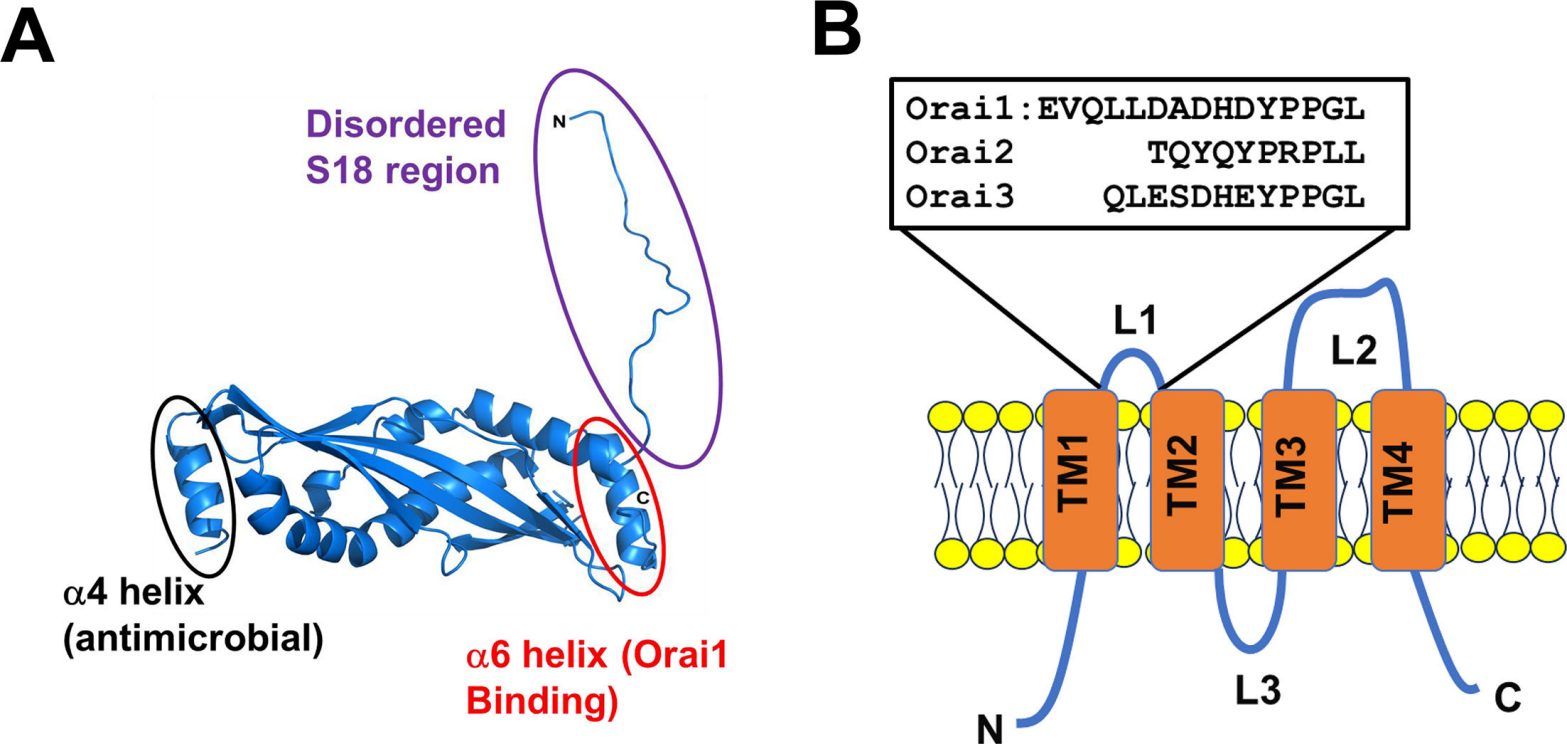
SPLUNC1 and Orai1. **(A**) Structure of SPLUNC1, with the disordered S18 region added in Pymol. The S18, α4 and α6 regions, along with their known functions, are highlighted. (**B**) 2-dimensional structure of Orai1, along with Orai1-3 extracellular loop 1.

SPLUNC1 is a 27-kDa secreted protein that can be glycosylated [4,5]. Human SPLUNC1 has an N-terminal secretory signal sequence (amino acids 1–19), disordered sequence, and then dubbed the S18 region, which binds to the epithelial Na^+^ channel [4,6,7] (Figure 1A). The ordered portion of SPLUNC1 contains six α-helixes and two β-sheets [7,8]. SPLUNC1 has been proposed to be a member of the tubular lipid binding protein superfamily [9] and exerts antimicrobial activity [10–12] through its α4 helix, which binds lipopolysaccharides [4,13,14]. The S18 region is conserved amongst primates but is absent from rodents. In contrast, the C-terminal α6 helix is well conserved across mammals, including primates and rodents [4,6,7], which may highlight its potential importance to mammalian physiology. Importantly, the α6 helix directly binds and inhibits the Orai1 Ca^2+^ channel (Figure 1A), which will be the main subject of this review [15,16].

## SPLUNC1 expression profile

SPLUNC1 is present in secretions from the upper and lower airways, saliva, ocular surfaces, tears and the middle ear [17–23]. With the advent of single-cell RNAseq (scRNAseq), SPLUNC1 was found to be most highly expressed in goblet cells and has been proposed as a marker of this cell type [24]. Similarly, the idiopathic pulmonary fibrosis (IPF) cell atlas indicated that SPLUNC1 (BPIFA1) was most highly expressed in MUC5AC-positive/goblet cells and was also expressed, albeit to a lesser extent, in club, ciliated and basal cells [https://www.ipfcellatlas.com/]. Consistent with SPLUNC1 being most highly expressed in terminally differentiated cells, Campos et al. previously found that SPLUNC1 expression significantly increased in human airway cultures as they became fully differentiated [17].

β-Agonists, which elevate cAMP, can increase SPLUNC1 expression [25]. Similarly, activation of toll-like receptor 2 (TLR2) and subsequent activation of mitogen-activated protein kinases (MAPKs) increase SPLUNC1 expression [26]. Here, TLR2, via MAPKs, induces phosphorylation of the c-jun transcription factor, leading to increased SPLUNC1 transcription. Th2 cytokines (e.g. IL13) diminish SPLUNC1 expression [27,28]. However, little is known about how SPLUNC1 is down-regulated in other diseases. Given the potential importance of SPLUNC1 not only as a biomarker of lung health, but also as a regulator of Orai1-mediated inflammation, more study is needed into how SPLUNC1 is down-regulated in disease. It will be interesting to see if there is a common pathway, or if it is disease-specific.

SPLUNC1 may be expressed elsewhere in the body. A cell atlas hosted by the Broad Institute indicated that SPLUNC1 was present at greater mRNA levels in the testes and the pituitary than in the lungs [https://www.broadinstitute.org/research-highlights-human-cell-atlas]. We previously reported that SPLUNC1 was expressed in the kidney and small intestine at the message level [6]. Interestingly, SPLUNC1 protein was detected in the urine of healthy normal subjects by mass spectrometry, confirming that SPLUNC1 was expressed in the nephron [29]. Some researchers found SPLUNC1 to be expressed in peripheral blood monocytes [30], whilst others did not detect it in peripheral blood neutrophils, monocytes or B/T cells [31]. SPLUNC1 has been detected in plasma by mass spectrometry. Thus, SPLUNC1 may (i) be secreted into plasma by circulating immune cells [https://v19.proteinatlas.org/humanproteome/blood/proteins + detected+in+ms], (ii) be secreted by underlying endothelia or (iii) reach the blood via epithelia. Moreover, SPLUNC1 is also expressed in epithelia lining the thymus [32,33]. Thus, immature T cells may be exposed to SPLUNC1. We have previously shown that SPLUNC1 can regulate Ca^2+^ signaling in epithelia and multiple immune cells [15]. Given the powerful control that SPLUNC1 can exert on the immune system, more experiments are needed to fully understand whether SPLUNC1 protein is present and/or active systematically.

## SPLUNC1 expression in chronic lung disease

SPLUNC1 levels change in many lung diseases (Table 1), and SPLUNC1 is an asthma and cystic fibrosis (CF) gene modifier [38,39]. SPLUNC1 protein levels in sputum inversely correlated with the frequency of exacerbations in both pediatric and adult CF patients, and patients with higher SPLUNC1 levels were admitted to the hospital less often [21,40]. Similarly, SPLUNC1 inversely correlated with the lung function in sputum from non-CF bronchiectasis patients [41]. SPLUNC1 message levels were up-regulated in CF patient tissues [31]. Neutrophil elastase is a key protease that is up-regulated in CF lungs and that drives tissue damage [42]. SPLUNC1 was rapidly degraded in CF sputum, and this degradation was caused primarily by neutrophil elastase [22,43]. It is likely that the CF lung tries to make more SPLUNC1 (up-regulated message) but is unsuccessful since SPLUNC1 is then degraded in the airway lumen.

**Table 1:** Summary of changes in SPLUNC1, Orai1 and SOCE in different pulmonary diseases.

Disease	Cellular SPLUNC1 levels	Secreted SPLUNC1 levels	Orai1 expression	SOCE	Notes
Asthma	↓	↓	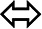	↑	No change in Orai1 expression, but increased STIM-Orai1 co-localization in human airways [34].
Bronchiectasis	?	↓	?	?	SPLUNC1’s impact on bronchiectasis pathogenesis has not been studied
CF	↑	↓	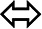	↑	No change in Orai1 expression, but increased STIM-Orai1 co-localization in human airways [34]. Enhanced Ca^2+^ entry in CF peripheral blood neutrophils [35,36].
COPD	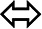	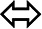	?	↑	Adducts from smoke can bind and inactivate secreted SPLUNC1 [37]. SOCE increased after exposure to cigarette smoke extract or e-cigarettes.
IPF	↓	?	↑	?	SPLUNC1’s impact on IPF pathogenesis has not been studied
PAH	↓	↓	↑	?	SPLUNC1’s impact on PAH pathogenesis has not been studied

SPLUNC1, short palate lung and nasal epithelial clone 1. SOCE, store-operated Ca^2+^ entry. STIM, stromal interacting molecule. PAH, pulmonary arterial hypertension. COPD, chronic obstructive pulmonary disease.

IL-13 is a typical Th2 cytokine that is up-regulated in asthma patients and is causal for airway disease [44]. We and others found that IL-13 significantly drives down SPLUNC1 expression levels in normal airway cultures [16,25]. Similarly, asthma patients with elevated IL-13 have reduced SPLUNC1 protein in their sputum. Reduced SPLUNC1 expression in asthma is associated with a hyperinflammatory phenotype, including increased airway smooth muscle contraction and abnormal eosinophil activity [16,39,45,46]. Moreover, it appears that elevated SPLUNC1 levels in the lung lumen are protective.

IPF is an interstitial lung disease characterized by excessive scarring of the lungs that leads to a progressive decline in lung function [47]. Here, Transforming Growth Factor beta (TGF-β)-induced fibroblast activation and conversion to myofibroblasts play a major role in driving disease progression [48]. Data mining of the IPF cell atlas suggested that SPLUNC1 message was reduced in IPF patient lung samples compared with healthy controls, while Orai1 was up-regulated in fibroblasts and myofibroblasts [https://www.ipfcellatlas.com/]. Interestingly, TGF-β can activate store-operated Ca^2+^ entry (SOCE)/Orai1. Moreover, inhibition of Orai1 reversed renal fibrosis [49,50]. Thus, it is tempting to speculate that abnormal SPLUNC1-Orai1 regulation contributes to IPF, but further studies will be needed to validate or refute these findings.

Pulmonary arterial hypertension (PAH) is a severe chronic disease characterized by abnormal and irreversible remodeling of the pulmonary arteries that leads to life-threatening elevations in blood pressure and right ventricular failure [51]. Indeed, PAH has up to a 55% mortality rate at 3 years post-diagnosis [52]. Orai1 has been established as a positive regulator of vascular remodeling [53,54], and Orai1 is up-regulated in the vasculature of PAH patients [54,55]. SPLUNC1 was significantly down-regulated in multiple PAH patient omics datasets compared with healthy controls [56]. Interestingly, this decrease in SPLUNC1 was confirmed by quantitative polymerase chain reaction (qPCR) and western blotting in the rat monocrotaline PAH model [57]. Moreover, Cl^−^ channels such as TMEM16A, which are Ca^2+^-activated, have also been implicated in vascular remodeling and are up-regulated in PAH patients [58]. Thus, it is tempting to speculate that reduced SPLUNC1 levels contribute to Orai1 and/or TMEM16A hyperactivity that drives vascular remodeling in PAH patients. Due to the poor outcomes of PAH patients, further investigation into how SPLUNC1 may contribute to Orai1 dysregulation in PAH is warranted.

Whilst sputum SPLUNC1 protein levels were reduced in asthma, CF and non-CF bronchiectasis [16,21,22,39,41], SPLUNC1 was unchanged in chronic obstructive pulmonary disease (COPD) patients at mRNA and protein levels (both cellular and secreted into sputum) [5]. In a second study, SPLUNC1 was up-regulated at the mRNA level and by immunostaining in COPD patients [59], but SPLUNC1 was not measured in their sputum. We have previously shown that adducts from combustible cigarette smoke bind and inactivate SPLUNC1 [37,60]. Indeed, it is possible that adduct binding to SPLUNC1 from tobacco smoke both protects it from proteolytic degradation and inactivates it. Thus, SPLUNC1 regulation, and its relation to lung function in COPD patients (who are typically ex- or current smokers), may be different from other airway diseases in never-smokers such as CF. Certainly, in future studies that correlate SPLUNC1 protein levels with different lung diseases, smoking status should be included as a possible confounder. Thus, across multiple lung diseases, we speculate that the lungs try to up-regulate SPLUNC1 levels to dampen down inflammation and that this may fail due to the presence of excessive neutrophil elastase that degrades SPLUNC1, IL-13, which reduces expression, or tobacco smoke, which binds and attenuates SPLUNC1 function.

## SOCE overview

**Figure 2:**
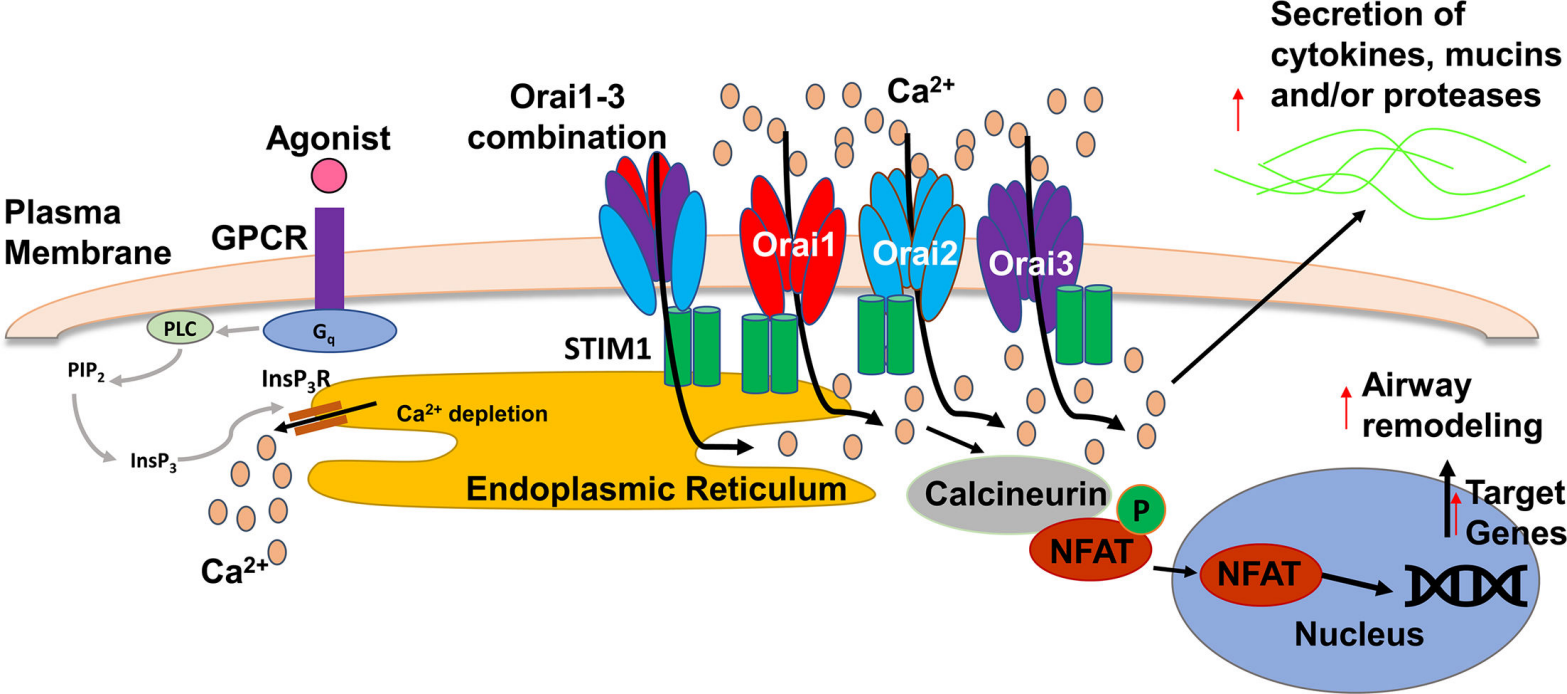
Overview of SOCE. Activation of G_q_-linked GPCRs causes phospholipase C (PLC) to hydrolyze PIP_2_ into InsP_3_. InsP_3_ activates the InsP_3_ receptor and stimulates Ca^2+^ release from the ER. STIM1 senses this Ca^2+^ depletion, oligomerizes to bind, oligomerize and activate Orai1. Ca^2+^ enters the cell via Orai1 and activates calcineurin, which dephosphorylates transcription factors, including nuclear factor of activated T cells (NFAT) in a cell-specific manner. These transcription factors then translocate to the nucleus facilitating inflammation and cellular remodeling.

SOCE is a ubiquitous signaling pathway that regulates many different physiological processes [53,61] (Figure 2). Activation of Gq-linked G-protein-coupled receptors (GPCRs) triggers the formation of inositol trisphosphate (InsP_3_) to initiate endoplasmic reticulum (ER) Ca^2+^ depletion. ER Ca^2+^ depletion then causes stromal interacting molecule 1 (STIM1) to rapidly relocate within the ER to ER–plasma membrane junctions, where it activates Orai1, 2 and/or 3 to initiate a second, amplifying wave of Ca^2+^ influx into the cytoplasm (Figure 2) [62]. SOCE occurs in multiple cell types and the only cell type that does not have SOCE is erythrocytes. ATP or UTP binding to purinergic receptors is a well-studied way of activating Gq, and subsequent SOCE in the airways [63]. Multiple groups have shown that SOCE increases ciliary beat frequency and activates Cl^−^ secretion via TMEM16A in airway epithelia. SOCE also initiates mucin secretion from goblet cells [63,64] and the secretions of cytokines and proteases from multiple cell types including neutrophils [65]. Thus, SOCE likely regulates mucin secretion, mucus hydration and mucus clearance from the airways [63], in parallel with regulating immune cell function and inflammation.

## Orai family members

The Ca^2+^ current that underlies SOCE was initially called I_CRAC_ for Ca^2+^ release activated Ca^2+^ current [66]. Orai1 was the first channel to be identified that carries I_CRAC_ [67]. Orai2 and Orai3 were subsequently identified. The complexity of Ca^2+^ homeostasis is further underscored by the observation that non-SOCE Ca^2+^ influx also occurs, which can be carried by ligand-gated ion channels (e.g. P2X purinoceptors) and/or voltage-gated Ca^2+^ channels [68]. For example, SOCE, P2X purinoceptors and transient receptor potential canonical (TRPC) channels have all been shown to influence Ca^2+^-dependent epithelial ciliary beating but are likely activated by different physiological and/or pathological stimuli [68].

Orai1, 2 and 3 have intracellular N- and C-termini, four transmembrane membrane (TM) spanning domains (TM1-4), one intracellular loop and two extracellular loops (Figure 1B). Orai1 has a theoretical molecular weight of 32 kDa but can be glycosylated and has been detected as an ~43 kDa band by western blotting [69]. Orai1 can exist as a full-length Orai1α (301 amino acids) or Orai1β, which lacks 64 N-terminal amino acids [70]. Orai1-3 have ~50% homology; the N- and C-termini and loops differ in sequence and length, while there is minimal sequence variation within the TM [70]. Orai1 is a hexamer [71], and Orai2 and Orai3 also form hexamers [72].

Orai1 has been well studied in airway smooth muscle [73,74], and we have previously shown that Orai1 is expressed in non-polarized primary airway epithelia [75]. More recently, immunohistochemistry has shown Orai1 to be expressed throughout the lungs [34]. Orai1 is also expressed in airway-relevant immune cells including, but not limited to, neutrophils and alveolar macrophages. scRNAseq data show that it is differentially expressed but generally present throughout the airways [https://www.broadinstitute.org/research-highlights-human-cell-atlas].

## Orai1/SOCE and transcription factors

A defining feature of I_CRAC_ is that it is inactivated as cytosolic Ca^2+^ increases (Ca^2+^-dependent inactivation) [76]. This Ca^2+^ inactivation contributes to Ca^2+^ oscillations, which, in turn, influence the activation of transcription factors and gene expression [72]. SOCE activation leads to a signal cascade that can activate multiple transcription factors in a cell-type-specific fashion [61]. Perhaps, the best described SOCE-sensitive transcription factor is NFAT. Increases in SOCE activate the phosphatase calcineurin, which culminates in dephosphorylation of NFAT family members, including NFAT1-4 [61], pro-inflammatory transcription factors that enter the nucleus [61,77] (Figure 2). However, other transcription factors have also been linked with activations of I_CRAC_/SOCE, including cAMP-response element binding protein, which can be activated by SOCE/calmodulin-dependent protein kinases (CaM kinases) [78]. Similarly, c-myc has been shown to be activated by SOCE in B cells, and c-fos was activated by SOCE in airway epithelial cultures [79,80]. SOCE can also regulate NF-κB, which, like NFAT, is involved in pro-inflammatory responses. Orai1’s regulation of NF-κB has been demonstrated in T cells [81] and osteoblasts [82]. Thus, Orai1/SOCE exerts significant effects on transcription in a cell-type-specific manner.

## Orai1 and lung disease

Goblet cell metaplasia is a form of epithelial mesenchymal transition (EMT) that is common in many lung diseases, including asthma, CF, COPD and non-CF bronchiectasis. Here, the number of ciliated cells diminishes, and the number of goblet cells increases, which leads to mucus hypersecretion and increased mucus plugging that prevents airflow [83]. Healthy airways typically exhibit a ratio of 80:20 ciliated cells to goblet cells, which can switch to 20:80 in CF and other muco-obstructive diseases. While SOCE’s role in EMT is well established, SOCE in goblet cell metaplasia is not fully understood. A number of transcription factors are associated with goblet cell metaplasia, including STAT6, FOXA2 and SPDEF [84]. STAT6 signaling is Ca^2+^-sensitive [85,86]. However, Orai1/SOCE’s role in activating these transcription factors during goblet cell metaplasia has not been studied.

Ca^2+^ influx/Orai1 activity is defective in airway smooth muscle from asthma patients and in murine asthma models [87,88]. Similarly, several groups have demonstrated that Ca^2+^ signaling is elevated in CF neutrophils and airway epithelia [35,36,89,90]. Perhaps surprisingly, despite the breadth of information on Orai1 and the immune system and smooth muscle [91], little is known about Orai1 expression in the lungs. We recently studied paraffin-embedded asthma, CF and healthy control lungs. We found that Orai1 was present at similar levels at the mRNA and protein levels across the three groups. However, Orai1 was expressed at greater levels in luminal immune cells and submucosal glands relative to the airway epithelia [34]. STIM1 must bind to Orai1 and organize Orai1 into puncta for SOCE to occur (Figure 2). In lungs from asthma and CF patients, STIM1-Orai1 co-localization was significantly increased, which is indicative of more Orai1 activation [34]. In contrast, STIM1-Orai1 co-localization was not altered in the alveolar regions, which is consistent with neither asthma nor CF being an alveolar disease. We also used super-resolution microscopy (~35 nm resolution) to identify Orai1 puncta and observed a significant increase in STIM1 and Orai1 puncta size in CF and asthmatic pulmonary immune cells relative to cells from normal lungs [34]. Taken together, these data strongly indicated that Orai1 was more active in asthma and CF than in normal lungs. Thus, we posit that Orai1 is an important, but under-appreciated, player in pulmonary inflammation that is convergent for multiple inflammatory pathways from both the innate and acquired immune systems. It will be interesting to use this approach to look for altered Orai1 activation in other lung diseases. Moreover, we propose that STIM1-Orai1 co-localization (Orai1 activation) can be used as a biomarker of inflammation in asthma, CF and other lung diseases.

### Orai1 therapeutics for the lungs

There are opposing views on how Orai1/SOCE should be modulated to treat lung disease. Some researchers propose activating SOCE, while others propose inhibiting it. Ultimately, the clinical effects may be due to drug efficacy, mechanism of action and/or route of administration. However, the influence that SOCE can exert on the lungs should carefully be considered before moving into human clinical trials.

#### The case for Orai1 activation

It has long been proposed that activation of SOCE will be beneficial for the treatment of CF lung disease since it may activate the TMEM16A Cl^−^ channel. Through the lens of an epithelial biologist, this makes sense since TMEM16A activation will increase Cl^−^ secretion into the lung lumen, leading to an increase in mucus hydration – a major defect in CF and other airway diseases. However, previous attempts at activating TMEM16A in CF patients failed clinically [92]. There is excessive neutrophil influx into CF lungs, and these cells typically lyse within 48 h, releasing ~10 mM ATP per cell into the lung lumen. Indeed, ATP and its metabolite AMP are an order of magnitude greater in concentration in CF compared with normal sputum [93], suggesting that ATP-activated SOCE is already active in CF airways. More recently, Genovese et al. have proposed to activate TMEM16A by potentiating IP3 activity and stimulating SOCE [94]. While they have tested this approach in cell lines, data have not yet been published on disease-relevant animal models.

A broad-spectrum increase in SOCE may have adverse effects since it could be highly pro-inflammatory and/or induce EMT. That is, in addition to activating TMEM16A, it could also stimulate secretion of mucins, proteases and cytokines, and trigger the activation of pro-inflammatory transcription factors that drive/maintain goblet cell metaplasia. Thus, since Orai1 may be causal for maintaining inflammation in the CF lung, potentiation of Orai1/SOCE in CF should be approached with caution. Perhaps, direct stimulation of TMEM16A without affecting SOCE, as proposed by Enterprise Therapeutics, may be a safer approach since it may increase airway hydration without increasing the secretion of macromolecules (e.g. cytokines, mucins and proteases) or inducing EMT [95].

#### The case for Orai1 inhibition

Conversely, inhibition of Orai1 has been proposed as a therapeutic for asthma, CF, acute respiratory distress syndrome (ARDS) and other lung diseases [53,91,96]. Here, inhibition of Orai1/SOCE is predicted to reduce inflammation and/or cellular remodeling by preventing the secretion of cytokines, mucins and proteases and/or reduce activation of pro-inflammatory transcription factors, leading to reduced cellular remodeling. Calcimedica has developed Auxora, a small molecule Orai1 antagonist that has been tested on people with ARDS caused by COVID [97,98]. Auxora has been delivered intravenously and has shown significant improvements in COVID/ARDS patients, suggesting that inhibition of Orai1 may be beneficial for treating lung disease.

### SPLUNC1 regulates Ca^2+^ signaling in the lungs

**Figure 3:**
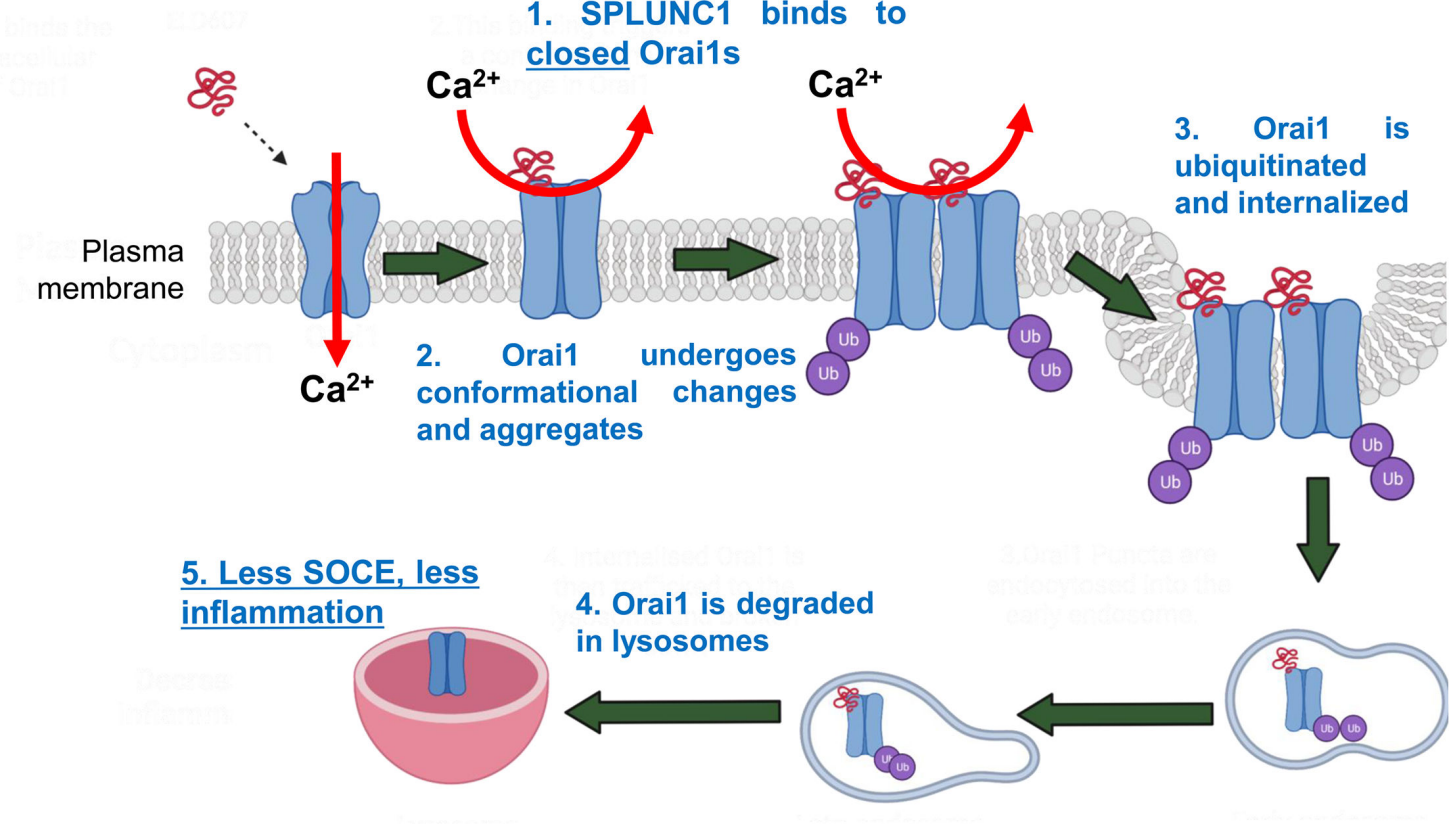
Model of SPLUNC1-induced Orai1 inhibition/degradation. (**A**) Small-molecule pore blockers prevent Ca^2+^ conductance but do not affect Orai1 internalization. (**B**) SPLUNC1 and SPLUNC1 peptidomimetics such as α6 and ELD607 induce conformational changes in Orai1 that lead to ubiquitination, internalization and degradation. Here, the reduced number of Orai1 channels causes the decrease in SOCE.

We previously found that SPLUNC1’s C-terminal α6 region directly binds and inhibits Orai1 [16]. SPLUNC1 induces a confirmational change in Orai1 that allows the ubiquitin ligase NEDD4.2 to bind and ubiquitinate Orai1, leading to increased Orai1 internalization [99,100] (Figure 3). In support of this, a hexameric Orai1 concatamer that is joined through its N- to C-termini was not internalized by SPLUNC1 [99,101]. We speculate that this hexamer was physically constrained and could not interact with the intracellular ubiquitin ligases needed to drive Orai1 internalization, but more experimentation will be needed to fully understand this phenomenon. We cannot exclude the possibility that SPLUNC1 directly blocks Orai1 conductance, but based on the onset of inhibition (~1–3 h), we speculate that SPLUNC1 works primarily by reducing the number of channels at the plasma membrane. SPLUNC1 interactions with other divalent cation channels have not been studied, but we previously found that a SPLUNC1 peptidomimetic neither bound to L-type Ca^2+^ channels nor interacted with Orai2 or Orai3 [102]. However, whether SPLUNC1 can bind to TRP channels remains to be determined.

SPLUNC1 significantly affects airway biology, namely by exerting immunoprotective/anti-inflammatory effects against both Th1 and Th2 inflammation [12,15,27,103]. These effects are most likely explained by SPLUNC1’s ability to inhibit Orai1. SPLUNC1^–/–^ mice have more neutrophilia in their lungs after bacterial *Pseudomonas* a*eruginosa* infection than their wildtype littermate controls, indicating that SPLUNC1 plays a key role in modulating inflammation [12,46]. Similarly, SPLUNC1^–/–^ mice up-regulate Orai1 protein and have an exuberant response to house dust mite exposure [15]. Moreover, specific inhibition of Orai1 by a SPLUNC1-derived peptide down-regulated Orai1 and resolved inflammation in these mice [15,102]. Complete loss-of-function Orai1 mutations are immunosuppressive, while ~70% inhibition of Orai1 does not cause an immunosuppressive phenotype [104], and SPLUNC1-mediated inhibition of Orai1 typically only reaches ~50% inhibition and is immunoprotective. Orai1 is certainly regulated physiologically; it is inactive until engaged by STIM1, which is modulated by several other proteins. Thus, one possible explanation is that SPLUNC1 is a physiological regulator of Orai1, which may be part of a larger network of Orai1 regulators [105], leading to a nuanced regulation of Orai1 that is immunomodulatory rather than immunosuppressive. Importantly, this established interaction between SPLUNC1 and Orai1 predicts that SPLUNC1 is much more than a biomarker of lung disease. However, further investigation will be needed to fully demonstrate a causal relationship between SPLUNC1, Orai1 and lung disease. Moreover, it will be interesting to determine if SPLUNC1’s anti-Orai1 effects extend to other tissues where SPLUNC1 is detected.

### Development of SPLUNC1-based Orai1 modulators for the treatment of lung disease

Due to neutrophil elastase-mediated degradation, SPLUNC1 protein is highly unstable in CF patient sputum [22]. Similarly, the α6 peptide was rapidly degraded in neutrophil elastase purified from CF sputum [102]. To circumvent SPLUNC1’s inability to regulate Orai1 in the highly proteolytic CF lung environment, we generated a protease-resistant SPLUNC1/α6 peptidomimetic called ELD607. This peptide was more stable in neutrophil elastase from CF patients and in ARDS patient airway secretions, and was 14 times more potent than SPLUNC1/α6 [102]. ELD607 was also size-optimized and ~30% smaller than α6, which increases aerosol delivery efficiency since more peptide is delivered per inhalation.

The majority of Orai1 antagonists occlude the channel pore, preventing Ca^2+^ from permeating the channel [62,106], while ELD607 binds specifically to Orai1’s first extracellular loop (Figure 3) [102]. Allosteric channel modulators have previously been described. For example, Inh_172_ is an allosteric CF transmembrane conductance regulator antagonist, which causes a conformational change that prevents Cl^–^ conductance [107]. Similarly, we propose that ELD607 is an allosteric Orai1 modulator that acts as a partial antagonist of Orai1. Importantly, ELD607 has an identical mechanism of action as SPLUNC1 (Figure 3), and we propose that ELD607 will restore SPLUNC1’s immunomodulatory functions to CF and other airways [99,108].

To evaluate ELD607’s efficacy, we first explored a bacterial pneumonia/ARDS model. We infected mice intratracheally with *P. aeruginosa* or *Staphylococcus aureus* variants, followed by inhaled delivery of ELD607 or vehicle. ELD607 alone was not antimicrobial, indicating that bacterial clearance was caused by immunomodulation rather than by direct antibiotic effects. Increased lung neutrophil levels causes lung damage and decreases survival [109]. ELD607 reduced neutrophilia and neutrophil elastase levels. Similarly, ELD607 significantly reduced markers of lung damage, as well as several cytokines, including IL-6, TNFα, IL-1β, KC, MIP-2 and IL-17, and increased blood arterial O_2_ levels and survival. Depletion of alveolar macrophages, but not depletion of neutrophils, abolished ELD607’s protective effects, indicating that ELD607 clears bacteria from the lungs by influencing macrophage activity. Macrophages are plastic, and whether ELD607 (and/or SPLUNC1) influences macrophage phenotype remains to be determined. Due to the rise in antibiotic-resistant bacteria, working via the immune system is a desirable drug characteristic since it is less likely to cause antibiotic resistance. Non-antibiotic approaches may offer an alternative to standard of care antibiotics for both acute and chronic bacterial infections.

Inhaled ELD607 did not cross the epithelial barrier and was retained in the lungs, suggesting that the lung lumen is its primary site of action. Since ELD607 was beneficial in the presence of high levels of bacteria, it was not immunosuppressive and, more importantly, was safe. However, additional chronic studies will be required in relevant disease models to determine whether ELD607 influences the acquired immune system. Importantly, since ELD607 was derived from SPLUNC1 and since (i) global SPLUNC1 knockout mice are immunosuppressive and (ii) SPLUNC1 is immunoprotective in CF patients, we predict that chronic inhalation of ELD607 will be protective against bacterial infections.

## Conclusions

**Figure 4:**
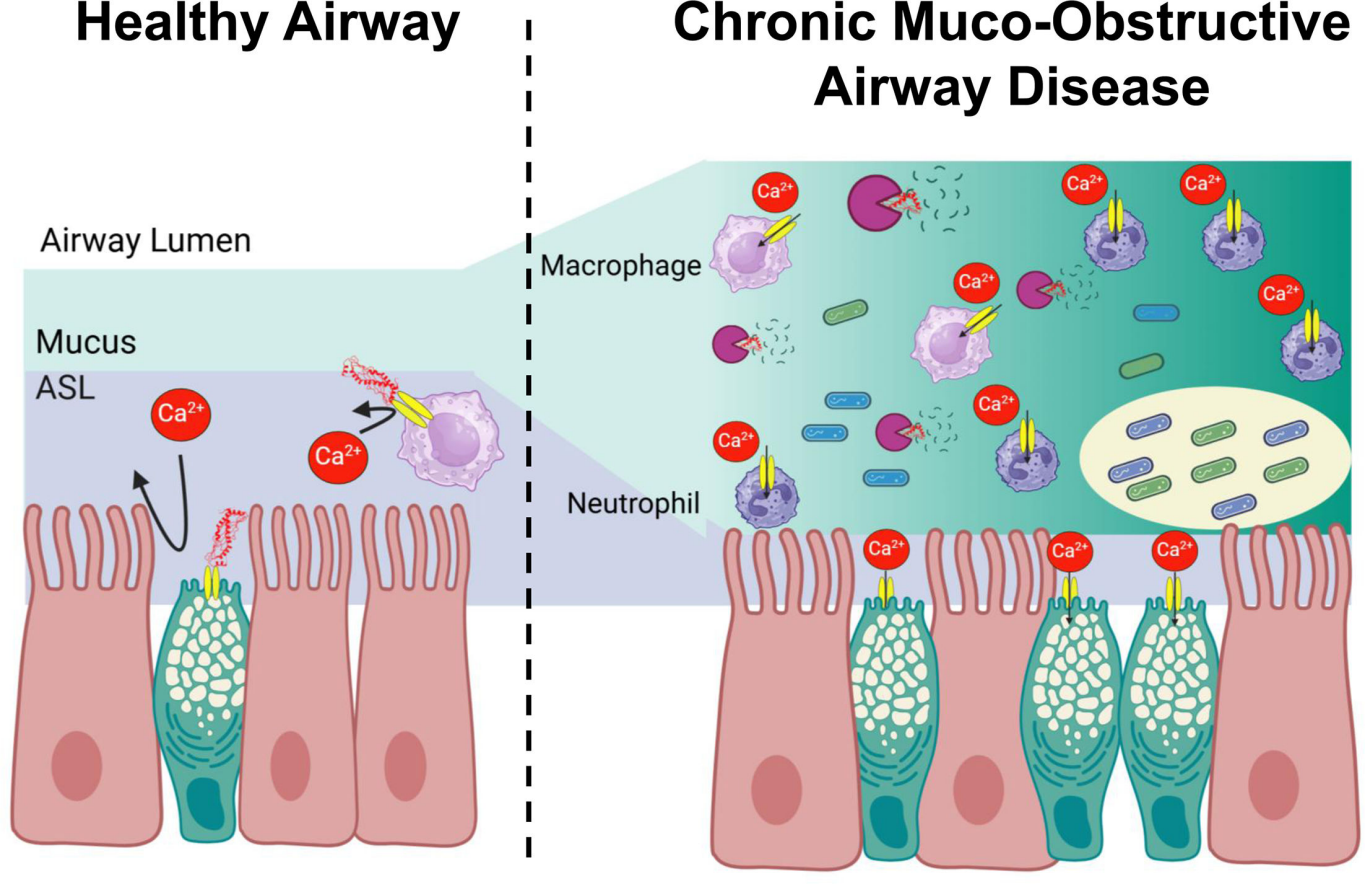
Working hypothesis for SPLUNC1 and innate defense of the airways. (**A**) In healthy airways, SPLUNC1 is secreted into the lung lumen, where it inhibits Orai1 in immune cells and/or airway epithelia lowering the chance of a pro-inflammatory response. (**B**) In chronic muco-obstructive airway disease, neutrophil elastase reaches a critical level and SPLUNC1 is degraded, leading to up-regulated Orai1 and unregulated/persistent inflammation that drives airway remodeling and fuels persistent neutrophilia.

We propose that SPLUNC1 is normally secreted into the lung lumen by goblet cells, where it can autocrinally/paracrinally inhibit Orai1 through its α6 region (Figure 4). Given that SPLUNC1 is a gene modifier/biomarker for multiple lung diseases, and since protease-resistant peptides of SPLUNC1 can fully reverse neutrophilia, we propose that with excessive inflammation, a tipping point is reached, SPLUNC1 is degraded by neutrophil elastase, and Orai1 activity is increased, leading to even more inflammation. Thus, we conclude that SPLUNC1 is a key secreted regulator of Orai1 that is critical for lung health.

**Perspectives**

- Orai1 is an established regulator of the immune system; decreased short palate lung and nasal epithelial clone 1 (SPLUNC1) protein is increasingly being recognized as being associated with poor lung health.
- We have found that SPLUNC1 binds to Orai1, setting in motion a chain of events culminating in Orai1 being degraded, which diminishes Ca^2+^ signaling that protects the lung.
- Future research should be directed at establishing a causal link between low SPLUNC1 and lung disease.

## Open Access

This article has been published open access under our Subscribe to Open programme, made possible through the support of our subscribing institutions, learn more here: https://portlandpress.com/pages/open_access_options_and_prices#conditional

## Abbreviations

ARDS: acute respiratory distress syndrome
BPIFA1: Bacterial Permeability Increasing Protein Family member 1
CF: cystic fibrosis
COPD: chronic obstructive pulmonary disease
EMT: epithelial mesenchymal transition
ER: endoplasmic reticulum
GPCR: G-protein-coupled receptor
IPF: idiopathic pulmonary fibrosis
MAPKs: mitogen-activated protein kinases
NFAT: nuclear factor of activated T cells
PAH: Pulmonary arterial hypertension
PLC: Phospholipase C
PLUNC: Palate Lung and Nasal epithelial Clone
SOCE: store operated Ca2+ entry
SPLUNC1: short palate lung and nasal epithelial clone 1
STIM1: stromal interacting molecule 1
TGFβ: Transforming growth factor beta
TLR2: toll-like receptor 2
TRPC: Transient receptor potential canonical channels
qPCR: Quantitative polymerase chain reaction
scRNAseq: single-cell RNAseq
